# Refuse in order to resist: metabolic bottlenecks reduce antibiotic susceptibility

**DOI:** 10.1038/s44320-025-00089-2

**Published:** 2025-02-18

**Authors:** Orestis Kanaris, Frank Schreiber

**Affiliations:** Division Biodeterioration and Reference Organisms, Department of Materials and the Environment, Federal Institute for Materials Research and Testing, Berlin, Germany

**Keywords:** Metabolism, Microbiology, Virology & Host Pathogen Interaction

## Abstract

O. Kanaris and F. Schreiber discuss a recent study by Link and colleagues (in this issue of *Molecular Systems Biology*), which reveals a connection between antibiotic resistance and primary metabolism in *E. coli*.

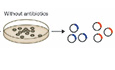

## Teaser

The growth of pathogenic bacteria in the host is a prerequisite for infectious diseases. Antibiotic drugs are used to impair bacterial growth and thereby treat infections. In turn, growth of bacteria is underpinned by their primary metabolism. Thus, it has long been recognized that the activity of antibiotics is determined by the metabolic state of cells. However, only recently researchers have begun to systematically interrogate the links between metabolism and resistance (Jiang et al, 2023; Lopatkin et al, 2021; Pinheiro et al, 2021; Schrader et al, 2021; Zhao et al, 2021). In their recent study, Lubrano and colleagues (Lubrano et al, 2025) apply an elegant CRISPR-based approach to the model bacterium *Escherichia coli* to systematically screen the effect of 15,120 mutations in genes that encode for 346 proteins which are required for growth of *E. coli* (also referred to as ‘essential proteins’). The authors identified a multitude of mutations that reduce the susceptibility against two antibiotics related to two very distinct chemical classes; the β-lactam antibiotic carbenicillin and the aminoglycoside gentamicin. Strikingly, the majority of the identified mutations are directly linked to primary metabolism. The work highlights the importance of metabolism in order to understand antibiotic resistance mechanisms and the ecology and evolution of antibiotic resistance. In addition, the work provides leads to design metabolism-based intervention strategies to mitigate antibiotic resistance.

**Figure 1:**
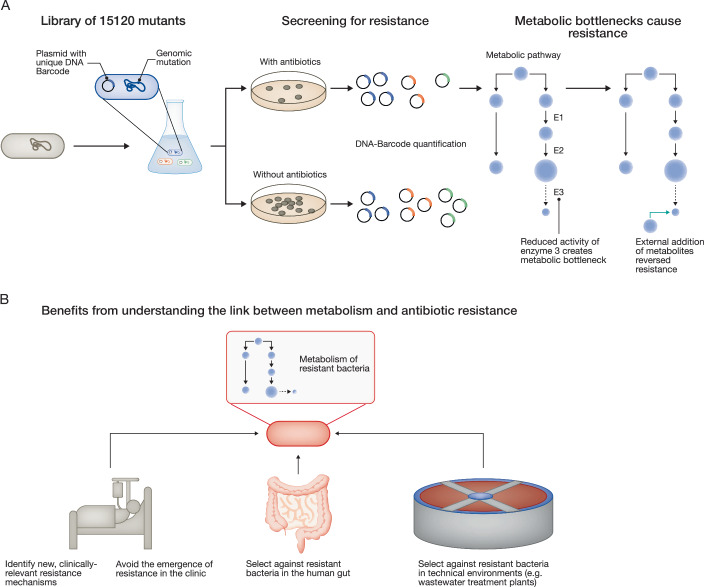
Systematic study of metabolic mutations that confer antibiotic resistance and potential application of the acquired knowledge. (**A**) Schematic representation of the methodology in Lubrano et al, 2025. A library of 15,120 mutants with one amino acid substitutions in one of 346 essential proteins per mutant was constructed. The whole library was cultured with and without any antibiotics in order to screen for resistant mutants. The abundance of each mutant was tracked by the quantification of unique DNA-barcodes in the mutants. Metabolomics was used to identify the effects of the mutations that reduced susceptibility to the antibiotics. Metabolic bottlenecks were identified to cause the changes in susceptibility. The external addition of the metabolites that were in shortage was shown to reverse the resistance in the mutants. (**B**) Potential applications of understanding the connection between metabolism and resistance. A better understanding of the metabolism of resistant bacteria can assist in combating infections, avoid resistance evolution during treatment, and shape ecological selection against antibiotic resistance in different environments.

The authors used the CRISPR-Cas9 system to generate a mutant library (Schramm et al, 2023). Each mutant in the library had one amino acid substitution in one of 346 essential proteins (Fig. 1A). In addition, each mutant was tagged with a unique DNA sequence barcode, which allowed mixing the mutants into a single culture and subjecting the mix to selection on antibiotics. This enabled the authors to track the abundance of each mutant by quantifying the unique sequence barcodes after selection on antibiotic-containing agar plates. The mutants were screened for an increase in their minimum inhibitory concentration (MIC) of at least 2-fold against carbenicillin and gentamicin. Most mutations that decreased antibiotic susceptibility were related to specific metabolic pathways. A MIC-increase to gentamicin was most frequently related to mutations in genes encoding for enzymes linked to central catabolic pathways. Specifically, mutants with low gentamicin susceptibility had mutations in genes encoding for (i) sugar transport into the cell, (ii) sugar catabolism by glycolysis, and (iii) biosynthetic enzymes involved in the synthesis of components of the respiratory chain. In contrast, mutations conferring increased MIC to carbenicillin occurred in genes encoding for enzymes related in purine nucleotide biosynthesis. Purines are building blocks of DNA and RNA. Thus, purine biosynthesis is a central anabolic pathway. In addition, purines are essential building blocks for major energy-carrying molecules including adenosine triphosphate (ATP) and guanosine triphosphate (GTP), which are involved in anabolic as well as catabolic reactions.

In the next step, the authors confirmed the observed effects by reconstructing a subset of selected mutations in the wild-type background. These mutant strains were also used to further understand the underlying mechanisms of reduced susceptibility. To this end, the authors quantified intracellular metabolites using metabolomics. This analysis revealed the accumulation of the substrates of the mutated enzymes as metabolites inside the cells, indicating that the mutations subsequently led to a reduced enzyme activity (Fig. 1A). Such reduced activity of a single step within a multi-step metabolic pathway is referred to as a ‘metabolic bottleneck’. The authors demonstrated that the MIC-increase was due to metabolic bottlenecks and not because of general reduced fitness. The external addition of the products of the metabolic pathways catalyzed by the mutated enzymes reversed the increase in MIC.

The finding that metabolism and antibiotic susceptibility are linked raises the question whether the observed mechanisms are relevant in the clinic. The observed decreases in antibiotic susceptibility are likely not relevant for categorizing a mutant to be resistant in the clinic for two reasons. First, the authors observed effects at rather small increases in antibiotic concentration. Metabolic mutants were observed for antibiotic concentrations up to 10×MIC, whereas the in-depth investigation in this study was focused on effects occurring at 2×MIC. Second, the observed effects on antibiotic susceptibility cannot be matched to clinical resistance because the authors did not use the standard medium that is commonly used for clinical resistance testing. The medium for standard clinical resistance testing is a nutrient-rich medium as opposed to the defined minimal medium used by the authors. In the nutrient-rich standard medium the observed metabolic bottlenecks might not occur and thus would likely not affect antibiotic susceptibility. This discrepancy underscores that current standard methods used to determine clinical resistance are not suitable to identify strains with decreased susceptibility underpinned by metabolic mechanisms. Thus, one future research direction is to investigate the role of metabolic differences between strains for contributing to clinically relevant resistance, considering realistic environmental conditions during infections and adapting the growth media to test for resistance in the laboratory accordingly.

Lubrano et al touched upon the issue of clinical relevance by showing that clinical *E. coli* isolates have the same metabolic bottlenecks as identified in their CRISPR screen. However, the metabolic bottlenecks were not directly linked to antibiotic susceptibility because addition of external metabolites did not affect susceptibility in these strains. This result highlights a limitation in our current understanding of the links between resistance and metabolism. The underlying mechanisms are likely multifactorial and highly dependent on interactions between the environmental conditions and the genetic background of different strains with a multitude of epistatic interactions affecting the outcome. Future research should therefore focus to uncover general principles (such as the ‘bottleneck hypothesis’) underlying the connection between resistance and metabolism, and then test whether they play a role under conditions relevant to infection.

Ultimately, changes in metabolism can impact the ecology and evolution of antibiotic resistance, both in the host and in the environment. The ecology of resistant strains can be affected by metabolism because metabolic differences may determine ecological niches, and thereby indirectly shape the selection of resistant strains in specific environments (Letten et al, 2021). Moreover, metabolic differences may shape the evolution of higher levels of resistance, whereby the decreased susceptibility conferred by metabolic differences may be a stepping-stone for the evolution of higher levels of resistance (as indicated by an experiment in the current study) (Levin-Reisman et al, 2017). These consequences of metabolic differences between resistant strains may in the future be used to devise metabolism-based, ecological intervention strategies to mitigate the emergence of resistance during treatment or to foster the removal of resistant strains from different eco-systems including the gut and wastewater (Fig. 1B) (Sanz-Garcia et al, 2023).

## References

[CR1] Jiang M, Su YB, Ye JZ, Li H, Kuang SF, Wu JH, Li SH, Peng XX, Peng B (2023) Ampicillin-controlled glucose metabolism manipulates the transition from tolerance to resistance in bacteria. Sci Adv 9:eade8582 36888710 10.1126/sciadv.ade8582 PMC9995076

[CR2] Letten AD, Hall AR, Levine JM (2021) Using ecological coexistence theory to understand antibiotic resistance and microbial competition. Nat Ecol Evol 5:431–441 33526890 10.1038/s41559-020-01385-w

[CR3] Levin-Reisman I, Ronin I, Gefen O, Braniss I, Shoresh N, Balaban NQ (2017) Antibiotic tolerance facilitates the evolution of resistance. Science 355:826–830 28183996 10.1126/science.aaj2191

[CR4] Lopatkin AJ, Bening SC, Manson AL, Stokes JM, Kohanski MA, Badran AH, Earl AM, Cheney NJ, Yang JH, Collins JJ (2021) Clinically relevant mutations in core metabolic genes confer antibiotic resistance. Science 371:eaba0862 33602825 10.1126/science.aba0862 PMC8285040

[CR5] Lubrano P, Smollich F, Schramm T, Lorenz E, Alvarado A, Eigenmann SC, Stadelmann A, Thavapalan S, Waffenschmidt N, Glatter T et al (2025) Metabolic mutations reduce antibiotic susceptibility of E. coli by pathway-specific bottlenecks. Mol Syst Biol 10.1038/s44320-024-00084-z 10.1038/s44320-024-00084-z PMC11876631 39748127

[CR6] Pinheiro F, Warsi O, Andersson DI, Lassig M (2021) Metabolic fitness landscapes predict the evolution of antibiotic resistance. Nat Ecol Evol 5:677–687 33664488 10.1038/s41559-021-01397-0

[CR7] Sanz-Garcia F, Gil-Gil T, Laborda P, Blanco P, Ochoa-Sanchez LE, Baquero F, Martinez JL, Hernando-Amado S (2023) Translating eco-evolutionary biology into therapy to tackle antibiotic resistance. Nat Rev Microbiol 21:671–685 37208461 10.1038/s41579-023-00902-5

[CR8] Schrader SM, Botella H, Jansen R, Ehrt S, Rhee K, Nathan C, Vaubourgeix J (2021) Multiform antimicrobial resistance from a metabolic mutation. Sci Adv 7:eabh2037 34452915 10.1126/sciadv.abh2037 PMC8397267

[CR9] Schramm T, Lubrano P, Pahl V, Stadelmann A, Verhulsdonk A, Link H (2023) Mapping temperature-sensitive mutations at a genome scale to engineer growth switches in Escherichia coli. Mol Syst Biol 19:e11596 37642940 10.15252/msb.202311596 PMC10568205

[CR10] Zhao XL, Chen ZG, Yang TC, Jiang M, Wang J, Cheng ZX, Yang MJ, Zhu JX, Zhang TT, Li H et al (2021) Glutamine promotes antibiotic uptake to kill multidrug-resistant uropathogenic bacteria. Sci Transl Med 13:eabj0716 34936385 10.1126/scitranslmed.abj0716

